# Moderate weight loss decreases lipedema-affected body fat mass in a woman who is lean with lipedema

**DOI:** 10.1210/jcemcr/luag018

**Published:** 2026-02-27

**Authors:** Giuseppe De Girolamo, Gordon I Smith, Richard I Stein, Thomas F Wright, Samuel Klein

**Affiliations:** Center for Human Nutrition, Washington University School of Medicine, St. Louis, MO 63110, USA; Department of Medical and Surgical Sciences, University of Foggia, Foggia 71122, Italy; Center for Human Nutrition, Washington University School of Medicine, St. Louis, MO 63110, USA; Center for Human Nutrition, Washington University School of Medicine, St. Louis, MO 63110, USA; Laser, Lipo and Vein Center, St. Louis, MO 63368, USA; Center for Human Nutrition, Washington University School of Medicine, St. Louis, MO 63110, USA

**Keywords:** insulin sensitivity, calorie restriction, adiposity, case report

## Abstract

Lipedema is a lipodystrophic disease characterized primarily by a disproportionate increase in lower body subcutaneous fat. Although moderate weight loss decreases lower body fat mass in women with obesity and lipedema, it is possible that this decrease is due to a reduction in normal subcutaneous fat, rather than lipedema-affected fat. We evaluated the effect of moderate (11%) diet-induced weight loss on body fat mass and distribution, assessed by dual-energy X-ray absorptiometry and magnetic resonance imaging, in a 56-year-old woman with lipedema who was normal weight (body mass index: 23.9 kg/m^2^) at baseline. Approximately 85% of the decrease in body weight comprised body fat. The relative reduction in upper body fat (abdominal subcutaneous, arm and trunk fat) was similar to the relative reduction in lower body (total leg fat and thigh subcutaneous fat). Accordingly, weight loss did not change the proportion of total body fat comprising leg fat (44.8% and 45.1% before and after weight loss, respectively) or arm fat (9.1% and 9.6% before and after weight loss, respectively). These data suggest weight loss decreases lipedema-affected adipose tissue and demonstrate the therapeutic effect of weight loss on body composition in women with lipedema even if they are normal weight.

## Introduction

Lipedema is a lipodystrophic disease characterized by bilateral, disproportionate lower body accumulation of subcutaneous adipose tissue with sparing of the hands and feet that occurs almost exclusively in women [1, 2]. Lipedema-affected adipose tissue is painful, tender and easy to bruise, which together with the disproportionate increase in adipose tissue mass typically causes impaired mobility, chronic pain, and reduced quality of life [1, 2]. Women with obesity who also have lipedema have increased inflammation and fibrosis in affected adipose tissue depots [3-6]; are more insulin sensitive than women who are obese without lipedema [3]; and are at a lower risk of developing metabolic abnormalities typically associated with obesity, including type 2 diabetes [7-9]. Although it is commonly believed that lipedema-affected fat depots are resistant to diet-induced weight loss [1, 10-13], we recently found that ∼10% weight loss in women with obesity and marked lower body lipedema caused the same relative reduction in abdominal subcutaneous fat as in subcutaneous thigh and total leg fat [3]. However, it is possible that the weight loss-induced decrease in lower body fat was due to a reduction in normal subcutaneous adipose tissue mass, rather than lipedema-affected adipose tissue because the participants had obesity.

Here, we present a case report that evaluated the effect of 11% weight loss on body composition (total body fat mass and body fat distribution) and metabolic function (plasma lipid profile, oral glucose tolerance, and whole-body insulin sensitivity assessed by using the hyperinsulinemic-euglycemic clamp procedure in conjunction with stable isotopically labeled glucose tracer infusion) in a woman with lipedema who had a normal body mass index and did not have excessive total body adiposity.

## Case presentation

A 56-year-old woman with upper and lower body stage 1 lipedema (ie, increased subcutaneous adipose tissue in arms and legs that bruises easily and is nodular by palpation [14]) was studied before and after moderate (∼11%) diet-induced weight loss. At baseline, the participant presented with disproportionate increases in both arm and leg fat masses, bilateral fat nodules in both legs, bilateral ankle cuffing, and nonpitting edema in both thighs and arms. In addition, the participant reported leg tenderness and pain and easy bruising in lipedema-affected areas. The participant completed a screening evaluation that included a medical examination and standard blood tests. The participant did not have any medical illnesses, other than lipedema, and was not taking any medications. Written, informed consent was obtained from the participant before she participated in this study, which was approved by the Washington University Institutional Review Board and registered in ClinicalTrials.gov (NCT03271034).

## Diagnostic assessment

### Body composition analyses

Total body, trunk, leg and arm fat masses, the android-to-gynoid fat ratio (in general, the android region is the area between the lower ribs and the pelvis and the gynoid region is the area below the pelvis to the upper thighs) and fat-free mass were assessed by using dual-energy X-ray absorptiometry (DXA, Lunar iDXA, GE Healthcare, Chicago, IL) according to the manufacturer's guidelines [15]. Intrahepatic triglyceride content and thigh subcutaneous, abdominal subcutaneous, and intra-abdominal adipose tissue volumes were determined by magnetic resonance imaging (3-T superconducting magnet, Siemens, Siemens, Erlangen, Germany) using AMRA Researcher (AMRA Medical AB, Linköping, Sweden).

### Oral glucose tolerance test

The participant completed a 2-hour 75-g oral glucose tolerance test (OGTT) after fasting for ∼12 hours overnight. Blood samples were collected before and 120 minutes after glucose ingestion to assess plasma glucose concentration by using an automated glucose analyzer (Yellow Spring Instruments Co, Yellow Spring, OH).

### Hyperinsulinemic-euglycemic clamp procedure

The participant was admitted to the Clinical Translational Research Unit at Washington University School of Medicine in the early evening on day 1 and given dinner (50% carbohydrate, 35% fat, 15% protein) at 1900 hours, which contained one third of her estimated daily energy requirements [16]. The participant fasted until 0700 hours on day 2 when a primed, continuous infusion of [U-^13^C]glucose (priming dose: 8.0 µmol/kg body weight, infusion rate: 0.080 µmol/kg body weight/min) was started and continued for 210 minutes. At 1030 hours, an insulin infusion was started, initiated by a 2-step priming dose (200 mU/m^2^ body surface area [BSA]/min for 5 minutes followed by 100 mU/m^2^ BSA/min for 5 minutes) and then continued at a rate of 50 mU/m^2^ BSA)/min. The [U-^13^C]glucose infusion was stopped at the initiation of the insulin infusion because of the expected decrease in endogenous glucose production [17]. Euglycemia (∼100 mg/dL [SI: ∼5.6 mmol/L]) was maintained by infusing 20% dextrose, enriched to ∼1% with [U-^13^C]glucose. Arterialized venous blood samples were obtained before beginning the tracer infusion and every 6 to 7 minutes during the last 20 minutes of the basal and insulin infusion periods to assess plasma glucose concentration (Yellow Spring Instruments Co) and glucose tracer-to-tracee ratio by using gas chromatography/mass spectrometry [18]. Whole-body insulin sensitivity (assessed as total glucose rate of disposal per kg fat-free mass) was calculated as previously described [19].

## Treatment

After baseline testing was completed, the participant began a balanced calorie deficit diet weight loss program supervised by a study dietitian and behavioral psychologist. The initial daily energy content of the diet provided 75% of estimated energy requirements, which was adjusted weekly as needed to achieve a 0.5% to 1% per decrease in body weight per week. Once the targeted 10% weight loss goal was achieved, energy intake was modified to maintain a stable body weight (<2% change) for 4 weeks before the study tests conducted at baseline were repeated. Accordingly, blood tests, body composition analyses, OGTT and the hyperinsulinemic-euglycemic clamp procedure were repeated 20 weeks after starting the diet intervention after a 10.9% reduction in the participant's body weight (Table 1).

**Table 1:** Subject characteristics before and after weight loss

	Before	After	Percent change	Normal reference range
**Body mass and composition**				
Body mass index	23.9 kg/m^2^	21.3 kg/m^2^	−10.9	—
Body weight	68.1 kg	60.7 kg	−10.9	—
Body fat mass	24.8 kg	18.5 kg	−25.4	—
Body fat	36.7%	30.6%	−16.6	—
Abdominal subcutaneous fat	6.2 L	4.4 L	−29.0	—
Intra-abdominal fat	1.9 L	1.2 L	−36.8	—
Thigh subcutaneous fat	10.5 L	7.7 L	−26.7	—
Total leg fat	11.1 kg	8.3 kg	−25.2	—
Total arm fat	2.3 kg	1.8 kg	−21.7	—
Trunk fat mass	10.5 kg	7.5 kg	−28.6	—
Android-to-gynoid ratio	0.7	0.6	−14.3	—
Intrahepatic triglyceride content	1.7%	1.7%	0	—
**Lipid profile**				
Triglyceride	48 mg/dL (0.54 mmol/L)	50 mg/dL (0.57 mmol/L)	+4.2	<150 mg/dL (<1.7 mmol/L)
HDL-cholesterol	71 mg/dL (1.84 mmol/L)	72 mg/dL (1.86 mmol/L)	+1.4	>50 mg/dL (>1.3 mmol/L)
LDL-cholesterol	153 mg/dL (3.96 mmol/L)	135 mg/dL (3.49 mmol/L)	−11.8	<100 mg/dL (2.6 mmol/L)
Total cholesterol	234 mg/dL (6.05 mmol/L)	217 mg/dL (5.61 mmol/L)	−7.3	<200 mg/dL (<5.2 mmol/L)
**Glucose control**				
Hemoglobin A1c	4.6% (27 mmol/mol)	4.8% (29 mmol/mol)	+4.3	<5.7% (<39 mmol/mol)
Fasting glucose	81 mg/dL (4.5 mmol/L)	81 mg/dL (4.5 mmol/L)	0	70-99 mg/dL (3.9-5.5 mmol/L)
OGTT 2-hour glucose	114 mg/dL (6.3 mmol/L)	96 mg/dL (5.3 mmol/L)	−15.8	<140 mg/dL (<7.8 mmol/L)
Fasting insulin	1.7 µU/mL (10 pmol/L)	2.2 µU/mL (13 pmol/L)	+29.4	2-25 µU/mL (12-150 pmol/L)
Glucose Rd	74 µmol/kg FFM/min	83 µmol/kg FFM/min	+12.3	—

Abbreviations: FFM, fat-free mass; glucose Rd, glucose rate of disappearance from plasma; HDL, high-density lipoprotein; LDL, low-density lipoprotein; OGTT, oral glucose tolerance test.

Values in parentheses are International System of Units (SI).

## Outcome and follow-up

Approximately 85% of the decrease in body weight was body fat. The relative reduction in total body fat (25.4%) was about 2.5 greater than the relative reduction in body weight (10.9%) (Table 1). The relative reduction in total body fat mass was similar to the relative reductions in upper body (ie, abdominal subcutaneous, arm and trunk fat) and lower body (total leg fat and thigh subcutaneous fat masses) (Table 1). Weight loss did not change the relative contribution of leg fat (44.8% and 45.1% before and after weight loss, respectively) or arm fat (9.1% and 9.6% before and after weight loss, respectively) to total body fat. Intrahepatic triglyceride content, fasting triglyceride, high-density lipoprotein-cholesterol, glucose and insulin concentrations, 2-hour OGTT plasma glucose concentration, and hemoglobin A1c were normal at baseline and did not change after weight loss (Table 1). The glucose disposal rate during the clamp procedures demonstrated that the participant had “high insulin sensitivity” both at baseline and after weight loss based on values we have previously observed in healthy lean participants [20]. Total and low-density lipoprotein-cholesterol at baseline were higher than the recommended normal range and decreased slightly after weight loss (Table 1).

## Discussion

We evaluated the effect of moderate (∼11%) diet-induced weight loss on body composition and metabolic function in a woman with lipedema who was not overweight/obese and had a normal body mass index. The assessment of subcutaneous fat in different depots, measured by using DXA and magnetic resonance imaging, demonstrated that the relative decrease in body fat was ∼2.5 fold greater than the relative decrease in body weight. Moreover, the relative reduction in fat mass was similar in depots affected by lipedema (ie, legs and arms) as in those not affected by lipedema (ie, abdominal fat). Accordingly, the percentage contribution of legs and arms fat to total body fat did not change with weight loss. In contrast, weight loss did not have clinically therapeutic effects on most cardiometabolic outcomes, including oral glucose tolerance, whole-body (primarily skeletal muscle) insulin sensitivity, intrahepatic triglyceride content, plasma triglyceride, and high-density lipoprotein-cholesterol concentrations, but did cause a small decrease in plasma total and low-density lipoprotein-cholesterol.

Our study has several limitations. First, we did not assess the effect of weight loss on pain and bruising in lipedema-affected adipose tissue or the effect of weight loss on mobility and physical function. Second, we did not assess dietary intake and macronutrient composition, which can affect the contributions from fat mass and lean mass to total weight loss. Third, our study is a case report that evaluated the effect of weight loss on body composition and metabolic function in a woman with lipedema and normal body weight; therefore, our findings might not apply to all people with lipedema.

In conclusion, the results from this case study demonstrate that diet-induced weight loss decreases lipedema-affected adipose tissue, even in women who are normal weight, and that moderate weight loss does not improve cardiometabolic outcomes that are already normal. These data support the therapeutic effect of weight loss in women with lipedema who are lean to decrease lipedema-affected adipose tissue.

## Learning points

- Moderate (11%) diet-induced weight loss reduces lipedema-associated fat depots with similar decreases in lipedema and nonlipedema affected depots.
- Moderate weight loss in people with lipedema does not improve cardiometabolic outcomes that are already normal at baseline.
- Weight loss has therapeutic benefits on lipedema-affected adipose tissue in women with lipedema who are normal weight.

## Data Availability

Original data generated and analyzed during this study are included in this published article.
